# City-scale distribution and dispersal routes of mycobiome in residences

**DOI:** 10.1186/s40168-017-0346-7

**Published:** 2017-10-04

**Authors:** Xinzhao Tong, Marcus H. Y. Leung, David Wilkins, Patrick K. H. Lee

**Affiliations:** School of Energy and Environment, City University of Hong Kong, Tat Chee Avenue, Kowloon, Hong Kong

**Keywords:** Mycobiome, Indoor built environment, Dispersal potentials, Distance-decay, Biogeography

## Abstract

**Background:**

Pathogenic and allergenic bacteria and fungi within the indoors can bring detrimental health effects on the occupants. We previously studied the bacterial communities found in households located throughout Hong Kong as well as the skin surfaces of the occupants. As a complementary study, here, we investigated the fungal communities (mycobiome) in the same residences and occupants and identified factors that are important in shaping their diversity, composition, distribution, and dispersal patterns.

**Results:**

We observed that common skin and environmental fungal taxa dominated air, surface, and skin samples. Individual and touch frequency strongly and respectively shaped the fungal community structure on occupant skin and residential surfaces. Cross-domain analysis revealed positive correlations between bacterial and fungal community diversity and composition, especially for skin samples. SourceTracker prediction suggested that some fungi can be transferred bidirectionally between surfaces and skin sites, but bacteria showed a stronger dispersal potential. In addition, we detected a modest but significant association between indoor airborne bacterial composition and geographic distance on a city-wide scale, a pattern not observed for fungi. However, the distance-decay effects were more pronounced at shorter local scale for both communities, and airflow might play a prominent role in driving the spatial variation of the indoor airborne mycobiome.

**Conclusions:**

Our study suggests that occupants exert a weaker influence on surface fungal communities compared to bacterial communities, and local environmental factors, including air currents, appear to be stronger determinants of indoor airborne mycobiome than ventilation strategy, human occupancy, and room type.

**Electronic supplementary material:**

The online version of this article (10.1186/s40168-017-0346-7) contains supplementary material, which is available to authorized users.

## Background

Individuals in urban societies spend most of their time indoors [1]. The indoor environment (also referred to as the built environment, BE) is also the habitat for microorganisms and represents a major interface of contact between humans and microbes [2]. In recent years, indoor microbiome has received much research interest, as organisms detected within BEs can be detrimental to human health [2–6]. Therefore, understanding factors that shape the indoor microbiome and its dispersal has great importance for occupants’ health and well-being.

Human skin itself is home to a diverse community of commensal microbiota, consisting of bacteria, fungi, archaea, and viruses [7–9]. Since the advent of high-throughput sequencing, bacteria, as the predominant skin colonizers, have thus far garnered tremendous attention in microbiome work [8, 10–12]. The diversity and composition of skin bacterial community are highly personalized [13] and site-specific [14]. Direct contact between human skin and indoor surfaces is a major route for microbial dispersal, and residential surfaces harbor unique skin bacterial signatures [15, 16]. In addition, occupants release particles into the surrounding environment and leave the indoor air with distinctive human-associated microbial fingerprints [17, 18].

While bacterial communities on skin are better understood, cutaneous microbial assemblages are not limited to bacteria, with fungi also occupying a wide range of skin niches [7]. Within fungal communities (mycobiome), *Malassezia*, *Rhodotorula*, *Debaromyces*, *Cryptococcus*, and *Candida* are among the most prevalent cutaneous taxa across different individuals and population groups [19, 20]. Direct shedding of fungal microbes from occupant clothing may exert an influence on the indoor mycobiome [21]. Furthermore, human occupancy and behavior appear to affect fungal richness and diversity within the BEs [18, 22]. In agreement with this, common skin fungal taxa, possibly originating from human occupants, are widely detected in indoor dust [2, 23–26], air [21, 27], or surfaces [22, 28] of different BEs.

In addition to the host-associated factors, indoor mycobiome also tends to be highly influenced by fungi from the outdoors [15, 22, 29, 30]. Outdoor microorganisms rarely cross significant geographic barriers through active dispersal [31], but the small size of microbes can facilitate their long-distance passive dispersal potentially with the help of air, water, and/or animals [31, 32]. The outdoor spatial variation in microbial diversity with geographic distance generates the distance-decay biogeographic pattern [32, 33], which is also encountered in indoor environments at different spatial scales [3, 21] as a function of outdoor dispersal [34].

To date, most indoor mycobiome studies have been largely limited to the western world, with only a few exploring the dispersals of mycobiome within the BEs [21, 34]. Furthermore, the reported fungal distance-decay pattern is either on a short geographic distance (400 m) within a housing complex [34] or in residences located across continents [3], with no information on a city-wide scale with different levels of urbanization and the associated factors driving the indoor biogeographic pattern. The mechanism(s) by which fungi disperse within BEs, and whether endemic fungal taxa exist in Asian households, are also unknown. In this study, the air, skin, and surface mycobiomes of 19 households distributed throughout Hong Kong (HK) were analyzed and compared with our previous bacterial work in the same households [15]. The objective is to determine whether, like bacteria, fungi can be transferred between surface and skin and, therefore, whether household surfaces harbor the human skin “fungal fingerprint.” Also, we aim to determine whether the indoor airborne mycobiome is strongly influenced by the local outdoor environment and whether households that are nearby share more taxa than those farther apart regardless of ventilation type and building design (i.e., demonstration of distance-decay relationships in the indoor airborne mycobiome).

## Methods

### Sample collection, DNA extraction, and sequencing

**Fig. 1:**
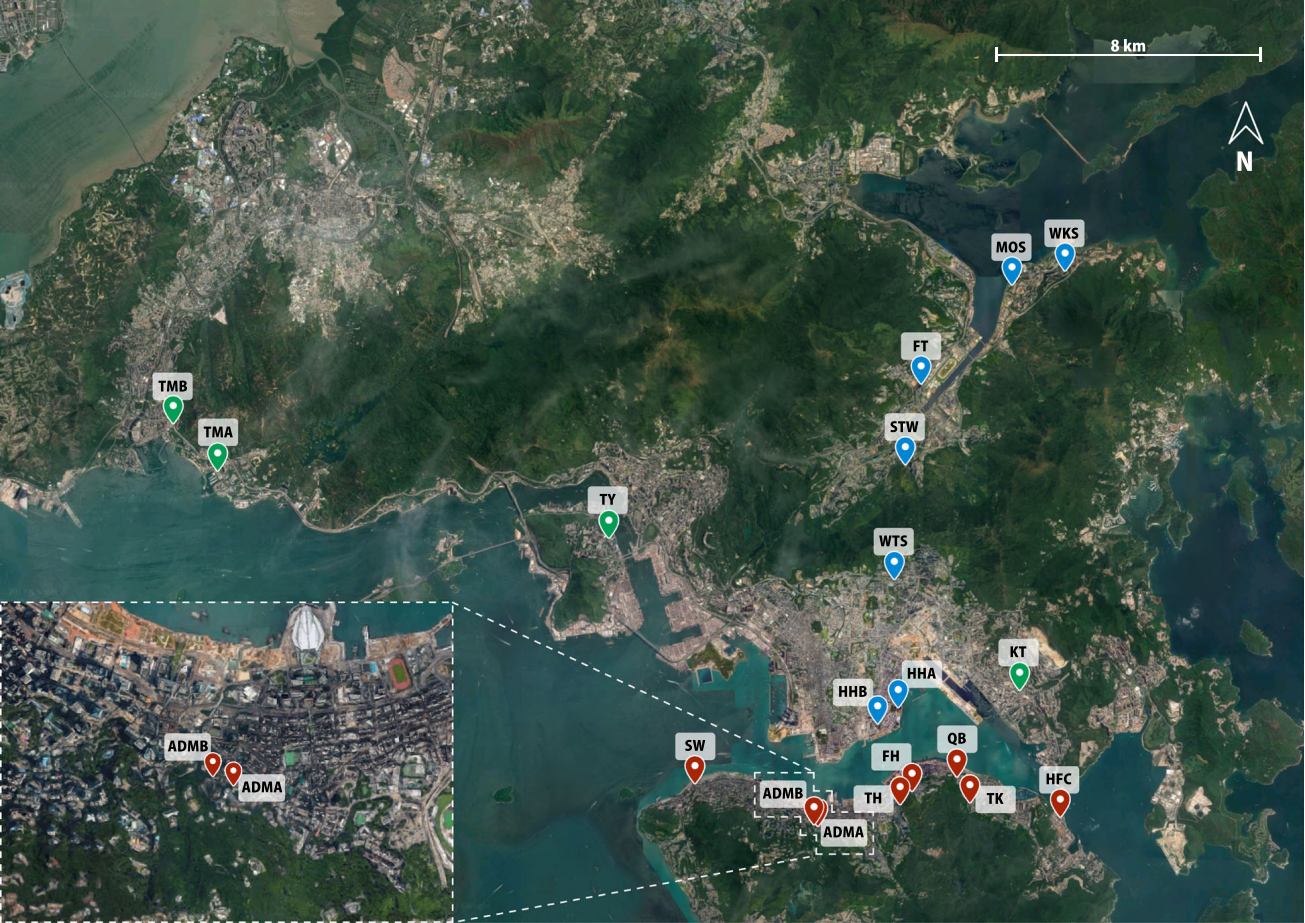
City-wide geographical locations of the 19 households. Households are separated into East-West (red markers, eight households) and North-South transects (blue markers, seven households), while the four households colored with green markers outside the two clusters were excluded from the directionality analysis. The locations of the households were plotted using Google Maps, and the household code names are indicated

Full details are provided in the supplementary method file (Additional file 1: Text S1). Air and surface samples were collected from 19 households distributed throughout HK (Fig. 1), and skin samples were sampled from 40 healthy occupants who lived in the residences. A single biological sample per sample type was collected from each home (428 in total). Genomic DNA was extracted, and fungal ITS1 region was amplified with the 18S*fw*/5.8S*rv* primer pair [14]. Libraries were prepared using the Illumina MiSeq Reagent Kit v2 and sequenced on a MiSeq platform to generate 250 bp paired-end reads.

### Bioinformatics

Forward and reverse reads were merged, trimmed, and filtered to a uniform length of 300 bp with an error rate of less than 0.5 error per read in USEARCH (version 9.0.2132) [35]. Dereplicated reads were clustered into operational taxonomic units (OTUs) at 97% identity following the UPARSE pipeline [36]. All sequences were first assigned taxonomy with the UNITE database [37] using the UCLUST algorithm in QIIME (version 1.9.1) [38], and a curated dataset [14] was then adopted to provide taxonomic information for reads that were unclassified at the genus rank with UNITE. Chimeras, singletons, and contaminants were discarded, and samples were rarefied to an even depth of 1058 reads per sample prior to the downstream analyses.

### Data analyses and statistics

Statistical analysis was implemented using the R framework (version 3.3.0). Differences in the relative abundance of a given genus between groups were determined using the Mann-Whitney (MW) [39] and Kruskal-Wallis (KW) [40] tests. Indicator species analysis was performed with the “indval” function in the R package “labdsv” [41]. The significances of different building and location factors on rarefied alpha- and beta-diversity of indoor mycobiome were assessed using analysis of variance (ANOVA) of the linear mixed-effects models in the R package “lme4” [42] and permutational multivariate analysis of variance (PERMANOVA) by the “adonis” function in the R package “vegan” [43], respectively. The tested metrics include observed number of OTUs, Chao1 [44], Shannon [45], and Simpson [46] for alpha-diversity and Bray-Curtis dissimilarity [47] and Binary Jaccard distance [48] for beta-diversity analyses. The factors considered above contained customized variables such as household occupancy and surface properties (details are provided in Additional file 2: Table S1). Spearman test was used to investigate the cross-domain bacterial and fungal alpha- and beta-diversity correlations. Mantel test [49] in the R package “ade4” [50] was applied to calculate the correlation between the geographic distance and the indoor bacterial or fungal community dissimilarity at three spatial scales, with Binary Jaccard as the community distance metric. Dispersals between different sample types within the households were predicted using the SourceTracker algorithm [51]. Indoor and outdoor air source tracking was carried out with HK indoor air samples as sinks and outdoor air samples from Beijing [52] and Berkeley [21] as the surrogate sources after performing closed-reference and open-reference OTU picking in QIIME. The sampling and sequencing information of the three studies used in source tracking are summarized in Additional file 3: Table S2.

## Results

### OTUs distribution

A total of 1501 fungal OTUs were retained after quality control of the sequencing data. Although some taxa were ubiquitous, the majority of OTUs (1371) were found in less than 10% of the samples, and 444 OTUs appeared only in one sample even after singleton OTU removal. The OTU rank-abundance curve shows a long tail of many low-abundance taxa (Additional file 4: Figure S1), which is also observed in another indoor mycobiome study [34]. Following rarefaction, skin and surface samples shared a larger proportion of their OTUs (145) than air and surface (54) or air and skin (38) (Additional file 5: Figure S2).

### Taxonomy and indicator species analysis

**Fig. 2:**
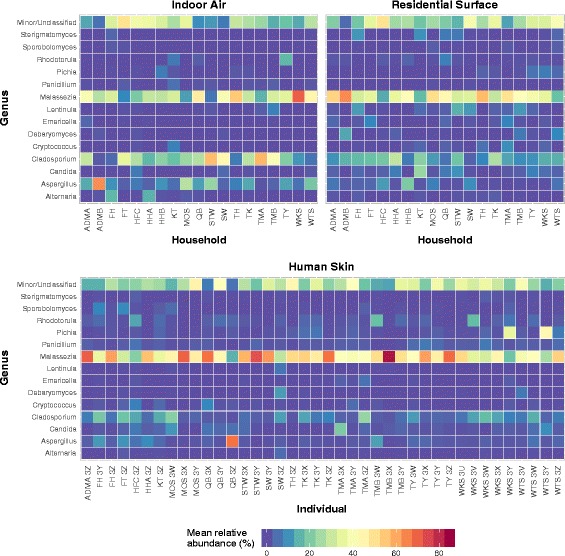
Mean relative abundance of the top 15 genera detected in samples across different households and occupants. The color intensity of the heatmap indicates a scale from 0 to 80%

*Malassezia* was the most prevalent genus across all three sample types, with a wide range of mean relative abundances from 6% to 83% (Fig. 2). Although *Malassezia* was more abundant on skin (KW post hoc test, *p* < 0.05 for comparisons between skin and air/surface), it also accounted for a large number of reads in the air and surface samples. Within surface samples, the relative abundance of *Malassezia* was significantly higher on those that were more frequently touched (MW test, *p* < 0.001, Additional file 6: Figure S3). No significant difference in the relative abundance of *Malassezia* was observed in air samples when considering the presence or absence of occupants (MW test, *p* = 0.955). Other less dominant taxa such as *Aspergillus* and *Cladosporium*, which are commonly found indoors, were especially abundant in air (KW post hoc test, *p* < 0.05 for comparisons between air and skin/surface, Fig. 2). At the species rank, several taxa uniquely associated with human activities in Asia were identified. These include *Aspergillus oryzae*, a filamentous fungus which is widely used in China and other Asian countries for fermentation [53]; *Cordyceps militaris*, a traditional Chinese medicine and folk tonic food in East Asia [54]; and *Auricularia polytricha* (cloud ear fungus) and *Lentinula edodes* (shiitake), commercially important mushrooms that are mainly distributed in the temperate and subtropical zones of Asia [55, 56].

Indicator species analysis identified some taxa that were noticeably more abundant in certain types of samples. For example, three OTUs of the mold genera *Cladosporium* and *Paecilomyces* served as unique indicator taxa of air samples. A small number of OTUs deriving from the common skin fungal members of *Malassezia*, *Penicillium*, and *Pichia* as well as an environmental species *Aspergillus niger* were the indicator taxa for skin samples. However, no indicator species was found for surface samples (Additional file 7: Table S3).

### Factors influencing fungal community diversity and composition

ANOVA was adopted to infer the influence of different factors on community alpha-diversity (Additional file 8: Table S4). After rarefaction, community richness (observed number of OTUs and Chao1) and diversity (Shannon and Simpson) metrics differed significantly by sample types, with skin showing the highest alpha-diversity among the three types of samples (*p* < 0.001 for all metrics). Specifically, air samples were significantly affected by household and relative humidity for all metrics except for Simpson when considering humidity as a factor, while room only showed influence on community richness. Factors (area, household, and individual) considered for skin samples all displayed significant effects regardless of the metric used. Area and room differed significantly for all metrics in surface samples except for Simpson when area was considered, and human occupancy did not affect the richness of surface community.

PERMANOVA was performed on rarefied Bray-Curtis dissimilarities (abundance-weighted community dissimilarity metric) and Binary Jaccard distances (membership-weighted community distance metric) between samples to estimate the effect of building and location factors on fungal community structure and composition. For the abundance-based dissimilarity metric (Table 1), household showed significant effects on all three sample types. Also, the community clustering effects on skin samples by individual (*p* = 0.001, *t* = 4.298) and surface samples by touch frequency (*p* = 0.001, *t* = 5.374) were the strongest among the factors considered within the same sample type. Furthermore, skin samples differed significantly when grouped by area, and surface samples by area and room. When only considering OTU presence/absence, factors including room on air community and deposition potential and human occupancy on surface community were significant, although the strength of clustering remained low (Additional file 9: Table S5). However, no significant difference was found in air communities between households with different ventilation systems (natural/mechanical) regardless of metrics used, suggesting that ventilation strategy played a lesser role in shaping the air mycobiome in the residences sampled.

**Table 1:** Bray-Curtis-based PERMANOVA test on factors affecting the indoor mycobiome

Factor	Air	Skin	Surface	All
Area		*p* = 0.003, *t* = 1.781	*p* = 0.001, *t* = 2.227	
Deposition			*p* = 0.054, *t* = 1.654	
Household	*p* = 0.001, *t* = 1.727	*p* = 0.001, *t* = 3.845	*p* = 0.001, *t* = 1.552	
Individual		*p* = 0.001, *t* = 4.298		
Occupancy	*p* = 0.287, *t* = 1.104		*p* = 0.081, *t* = 1.453	
Room	*p* = 0.396, *t* = 1.055		*p* = 0.001, *t* = 2.489	
Touch			*p* = 0.001, *t* = 5.374	
Type				*p* = 0.001, *t* = 12.790
Ventilation	*p* = 0.138, *t* = 1.448			

### Cross-domain comparison of bacterial and fungal communities

To explore the correlation between bacterial and fungal communities within each sample type, we combined the current fungal data with our previous work that analyzed indoor bacterial communities [15] of the same households and occupants. After rarefying to the respective sequencing depths, skin bacterial and fungal alpha-diversity correlated significantly and positively for both observed number of OTUs and Chao1 (Spearman, *p* < 0.001, rho = 0.290 for observed number of OTUs; *p* < 0.001, rho = 0.289 for Chao1, Additional file 10: Table S6), indicating that samples with a higher bacterial richness also tended to have a higher fungal richness. For surface samples, only the estimated total number of bacterial and fungal OTUs was significantly and positively correlated (Spearman, *p* < 0.001, rho = 0.301 for Chao1). However, no significant cross-domain alpha-diversity correlation was observed for air samples (Spearman, *p* = 0.252 for observed number of OTUs, *p* = 0.849 for Chao1).

The cross-domain beta-diversity correlation was performed based on the rarefied Bray-Curtis dissimilarities and Binary Jaccard distances, respectively (Additional file 11: Table S7). For both metrics, significant and positive bacterial-fungal community composition correlations were identified for all three sample types. Remarkably, skin samples (Spearman, *p* < 0.001, rho = 0.315, effect-size *r* = 0.291 for Bray-Curtis dissimilarity) showed the greatest positive correlations between bacterial and fungal community composition dissimilarities, and the effect size even doubled (Spearman, *p* < 0.001, rho = 0.237, effect-size *r* = 0.601 for Binary Jaccard distance) when only considering OTU presence/absence. However, regardless of the metric used, the effect size remained low for air and surface samples where the bacterial and fungal communities were moderately correlated.

### Distance-decay pattern

Our previous bacterial study [15] and current fungal work jointly revealed that household was the strongest factor that significantly shaped the composition and structure of the indoor airborne communities. Given that the households are distributed spatially across the city, here, we studied the correlation between the community dissimilarity (Binary Jaccard distance metric) and geographic distance for the two domains. On an overall city-wide scale without considering directionality (the longest point-to-point straight-line distance between households is 30 km), a weak but significant correlation was observed for the bacterial community (Additional file 12: Figure S4A), suggesting that households within close proximity of each other tended to share more bacterial taxa than households located further apart (*p* = 0.001, *r* = 0.033, Mantel test). However, this pattern was not observed for fungi (Additional file 12: Figure S4B), such that households closer together in geographical distance did not necessarily have a more similar indoor air mycobiome (*p* = 0.400, *r* = 0.002, Mantel test).

**Fig. 3:**
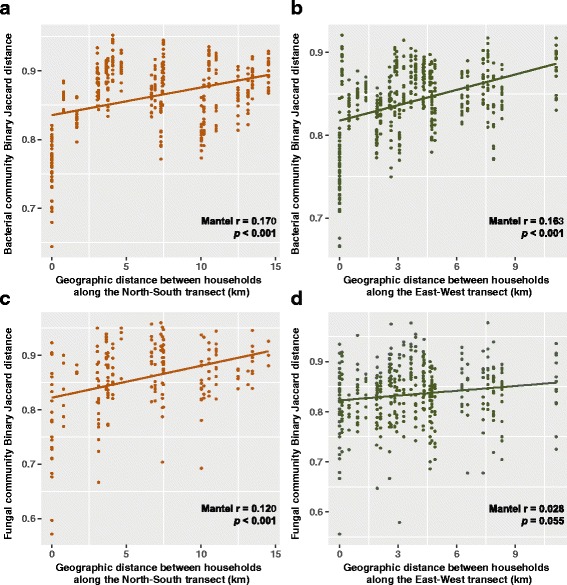
Distance-decay patterns of the indoor airborne bacterial and fungal communities for households along the North-South and East-West transects. Each point represents the bacterial or fungal community dissimilarity (based on Binary Jaccard distance) between two air samples at the indicated geographic distance for two households. The regression line between the bacterial or fungal community dissimilarities, and geographic distance is shown. Households located on the North-South and East-West transects for the bacterial (**a**, **b**) and fungal (**c**, **d**) communities, respectively

Given that the majority of households (15 of 19) are distributed along two directions (Fig. 1), we further separated these households into two subgroups, one from North to South (farthest apart is 15 km) which captures a large gradient of geographic landscape in HK (from dense city to rural area), and the other from East to West (farthest apart is 11 km) which has less variation in the level of urbanization. For air bacterial community, we observed significant positive correlations between community dissimilarity and geographic distance in both directions (*p* < 0.001, *r* = 0.170 for North-South transect; *p* < 0.001, *r* = 0.163 for East-West transect; Mantel test) (Fig. 3a, b). However, for air fungal community, significant result was only obtained for the North-South transect (*p* < 0.001, *r* = 0.120, Mantel test) and the community along the East-West transect (*p* = 0.055, *r* = 0.028, Mantel test) appeared to be more homogeneous (Fig. 3c, d).

### Dispersal pattern predictions

**Fig. 4:**
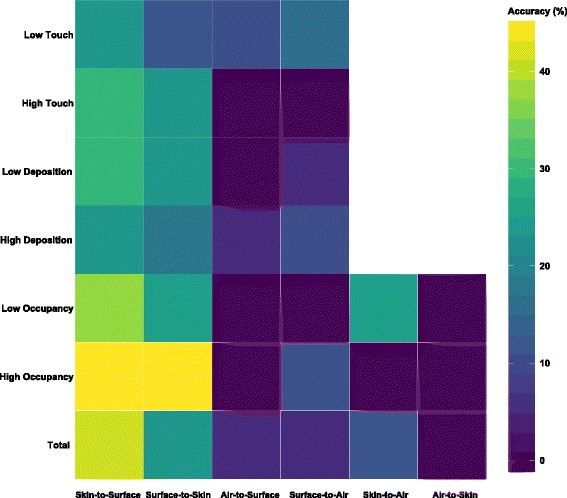
Heatmap based on SourceTracker prediction accuracy for the different dispersal routes. The color intensity of the heatmap indicates a scale from 0 to 40%. A prediction is considered successful only if the source community that contributes largest to the sink community is from the same household. The accuracy rate in percentage is the ratio between the number of successful predictions and total predictions. As an example, skin-to-surface indicates the dispersal route where skin samples act as the sources and surface samples are the sinks. For dispersal routes involving air or surface, occupancy level was tested. For routes involving surface either as a source or sink, the transfer was further evaluated based on high/low deposition potential and high/low touch frequency

The Bayesian SourceTracker technique [51] was used to assess dispersals between sample types within households. The highest prediction accuracy (~ 40%) was identified for the skin-to-surface route (Fig. 4), suggesting that two-fifths of household surfaces were imprinted with their corresponding occupant skin-associated fungal communities. In contrast, the prediction accuracy for the opposite direction (surface-to-skin route) was lower (~ 30%). However, all routes involving air either as a sink or source showed less than 12% accuracy, with the exception being dispersal from skin to air in households with low occupancy. Notably, no successful prediction (0%) was made for the air-to-skin route. Compared to our previous bacterial work, which used the same method to predict dispersal potentials [15], fungi appeared to be harder to disperse between skin and surface routes, where the average prediction accuracy for the bacterial community was ~ 70%.

**Fig. 5:**
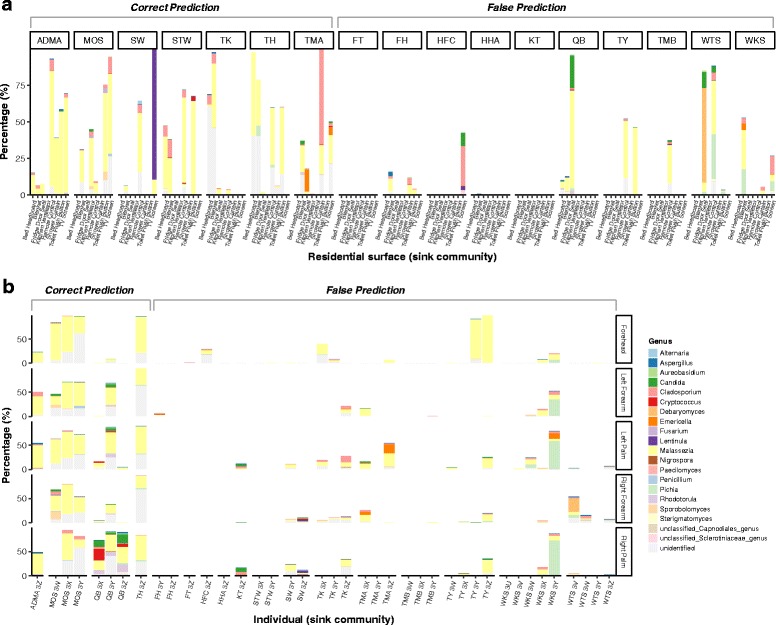
Contribution of the source genera to the corresponding sink community for each household. **a** The contribution of skin community to each residential surface (*x*-axis) within each household (households ADMB and HHB without skin samples were excluded from the analysis). Correct prediction indicates that SourceTracker is capable to correctly match the surface community to the corresponding occupant of the households, while false prediction indicates an incorrect match between the surface community and the occupant. The percentage refers to the total contribution of each genus on the five skin sites to each surface community. **b** The contribution of the surface community to each skin site (right *y*-axis) of the occupants (*x*-axis) within each household. The percentage refers to the total contribution of each genus on the eight different types of surfaces to each skin site

In order to further determine which OTUs could be transferred between sample types, and the extent to which the OTUs contributed to shaping the sink community, the relative contribution rates of the contributor OTUs to the correct sink communities were assessed for all households. The majority of contributor OTUs (genera) were identified to be the reciprocal sources between the two sample types analyzed (Fig. 5, Additional files 13 and 14: Figures S5 and S6), suggesting a large extent of bidirectional exchanges between the source and sink communities. However, a few contributor OTUs were identified to be the unique sources for either skin or surface community (Additional file 15: Table S8), suggesting that the dispersal of some OTUs only occurred in specific one-way routes.

### Comparison of HK indoor air mycobiome to outdoor air mycobiome of selected locations

To test the hypothesis that outdoor airborne fungal community was the major source for the indoor air mycobiome, outdoor air samples from Beijing [52] and Berkeley [21] were used as surrogate sources (Additional file 3: Table S2). Sequences in the three datasets were clustered into OTUs using two different approaches (closed-reference and open-reference workflows). SourceTracker was used to determine the proportion of outdoor mycobiome that made up the HK residential mycobiome. Although biases could be introduced as OTUs were more likely to cluster within its own dataset, the contribution of the surrogate sources on HK indoor air mycobiome remained high regardless of the OTU picking strategy (especially Beijing outdoor air samples with an average of ~ 50% contribution) (Additional file 16: Figure S7), supporting the hypothesis that the fungal communities in the outdoor air dominated the indoor airborne mycobiome.

## Discussion

### Dispersal between occupant skin and residential surfaces

Human skin is widely colonized with mycobionts including *Candida*, *Cryptococcus*, *Debaryomyces*, *Malassezia*, *Penicillium*, and *Rhodotorula* [14]. The interaction between skin and residential surfaces leads to close resemblance between the two respective microbial communities, and the surfaces can also be imprinted with human-associated microbiome signatures [15, 16, 57, 58]. In this study, skin fungal commensals dominated the skin samples, with many being unique indicator species of skin (Additional file 7: Table S3). Skin and surface samples exclusively shared a larger number of *Malassezia*-associated OTUs than those that were specific to surface (Additional file 5: Figure S2). In SourceTracker analysis (Fig. 4), the surface communities can be correctly matched to the corresponding occupant skin in over 40% of the households. *Malassezia*, comprising over one third of contributor OTUs (39/94), was not only a major source for surface communities in these correctly matched households, but also contributed to shaping the surface communities in households where predictions were incorrect (Fig. 5a), suggesting that the dispersal of skin fungi occurred in majority of the households. On the other hand, the surfaces in household KT appeared to be the vector rather than the recipient with *Candida*- and *Cladosporium*-associated OTUs on skin being identified as sourced exclusively from the surface community (Fig. 5 and Additional file 14: Figure S6). In addition, a small number of contributor OTUs identified on surfaces were the unique sources for skin communities (Additional file 15: Table S8). Therefore, fungi on occupant skin and household surfaces can be transferred bidirectionally. Given the relatively high abundance of *Malassezia* on frequently touched surfaces (Additional file 6: Figure S3), as well as the relatively high accuracy in predicting the dispersal from frequently touched surfaces to the occupant skin (Fig. 4), we contend that human contact is a major route for fungal dispersal between skin and household surfaces.

### Origin of the skin-associated fungi in residential air

Similar to surfaces, a high abundance of skin-associated taxa in the indoor air seems to imply strong dispersals from occupant skin. However, SourceTracker was less accurate when attempting to match the indoor airborne fungal communities to skin or surface samples (Fig. 4). In addition, only a few air samples contained taxa derived from skin or surface and no dispersal was observed in over half of the households (Additional file 13: Figure S5). Therefore, there is only weak evidence of dispersal from either skin or surface to air. This observation could be partly attributed to the fact that the sampling was conducted during winter, and the thicker clothes worn by the occupants may reduce the shedding of microbial particles from skin directly into indoor air [59]. However, given that a large proportion of skin-associated taxa, mostly *Malassezia*, were found in air, there are likely other sources that cause the indoor air to resemble the occupant skin community. In our previous bacterial work [15, 60], skin-associated taxa in the outdoor air have been identified as a major source of the indoor air. Here, SourceTracker results suggested that outdoor air similarly has a high potential to be the source of the indoor airborne mycobiome (Additional file 16: Figure S7). Therefore, it is possible that the majority of the skin-associated taxa in the indoor airborne mycobiome are sourced from the outdoor environment of HK (which is densely populated). Given the localization and endemism of the microbiome in the outdoor environment, having comparable local outdoor air samples from HK would be much more effective for source tracking of the indoor mycobiome [61], and this will be a promising avenue to pursue for future work.

### Contribution of the outdoor environment to the indoor mycobiome

In addition to the cutaneous fungi, indoor mycobiome contained relatively high abundance of environmental taxa. *Cladosporium*, identified as an indicator species of indoor air in this study, also accounted for a proportion of skin and surface communities. Despite this, the environmental taxa in indoor air community showed minimal dispersal tendency (Additional file 14: Figure S6), suggesting that indoor air is unlikely to be the major source of environmental taxa found in skin and surface communities. Furthermore, regionally endemic species such as *Auricularia polytricha* and *Cordyceps militaris* were only observed in surface and skin samples. *Lentinula edodes*, which was scarce in the air community according to our findings, was significantly enriched (*p* = 0.03, MW test) on surfaces with high deposition potentials. One reason could be that the relatively large spore size of these mushroom-forming fungi facilitated their deposition [62]. In addition, some fungal spores can adhere to abiotic surfaces without external influence [63], and the adhesion effect increases with the increasing surface roughness [64]. Therefore, it is possible that human skin and residential surfaces are major collectors of large spores that originated from the outdoors.

### The distance-decay biogeographic pattern

Architectural design is important for controlling the influence of outdoor air on the diversity and composition of the BE microbiome, with different ventilation strategies and characteristics shown to drive the variation in the indoor airborne community [65–67]. Here, the ventilation type (natural/mechanical) had no significant effect on either richness or composition of the indoor airborne mycobiome. This contrasts a recent study by Irga and Torpy [65] that showed naturally ventilated offices have a higher fungal diversity compared to those with mechanical systems. Households in the current study are chosen from a wide range of buildings spatially distributed on a city-wide scale, where differences in the nearby outdoor air have been reported to drive the variation in the indoor mycobiome of the BEs [34]. Therefore, the local environment could exert stronger influences on the indoor air mycobiome than building design when a broader scale is considered, as demonstrated by previous continental- [3] and global-scale [2] studies, where local environmental selection is the strongest determinant of household dust-associated mycobiome.

In this study, the geographic location of the households is the sole factor affecting the indoor mycobiome structure and composition. However, the distance-decay pattern of indoor airborne mycobiome was only observed in households located on the North-South transect (Fig. 3c). In contrast, the indoor bacterial community showed a weak but significant city-wide distance-decay pattern (Additional file 12: Figure S4A). These observations raise three questions: (1) What is driving the distance-decay pattern of the airborne bacterial and fungal communities? (2) Why do fungi and bacteria present different dispersal limitations? (3) If there is a correlation between fungal community and geographic distance in the North-South transect, why this pattern is not being observed in the East-West transect or even on a larger city-wide scale?

Fungi can disperse through sporulation [68], and a large number of propagules increase the chance for long-distance passive dispersal [31]. In addition, fungal spores are usually aerosolized as dry spores, while bacteria are generally trapped in liquid droplets before releasing into the air [69]. The relatively cooler and drier conditions in HK during the winter season [70] when the study was conducted may favor the dispersal of fungal spores relative to bacteria. Furthermore, given that particle detachment is proportional to the exposed particle surface area, and the removal force increases more rapidly than adhesion forces as particle size increases [71], the generally smaller size of bacteria compared to fungal propagules [18] is consistent with the theory that bacteria will detach under a higher airflow speed compared to fungi. Therefore, the lack of a city-wide scale distance-decay pattern in the indoor airborne mycobiome may be attributed to the physical capability of fungi in dispersing farther than bacteria during winter. Given that air is an important vector for the passive dispersal of small organisms [31], and variation in the landscape might cause spatial heterogeneity in the outdoor airborne microbial community [3], we suggest that air currents can promote microbial dispersal in the outdoor environment and further drive the biogeographic distribution of microbes in the adjacent BEs. The air mass passing through HK in winter is mostly sourced from the northwest (based on HYSPLIT air trajectory model) [70], and it likely plays a prominent role in driving the distance-decay pattern in the North-South transect, which also spans a range of geographic landscapes. On the other hand, the more homogenized mycobiome in the East-West transect might be due to the more uniform landscape in the air flow path (e.g., vegetation conditions, land-use type, and population density) (Fig. 1).

### Summary of the dispersal potentials for bacteria and fungi in households

**Fig. 6:**
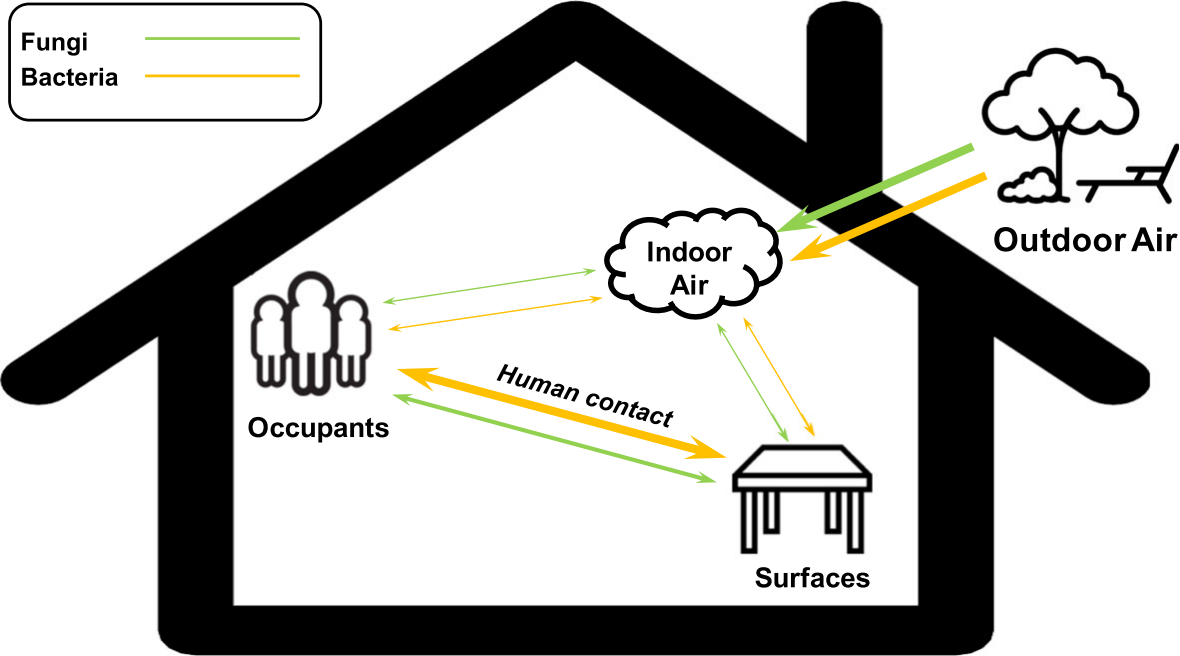
Schematic summary of the bacterial and fungal dispersal potentials for the different routes within households. Double-sided arrow depicts bidirectional dispersal of microbes. Line thickness is proportional to the strength of dispersal potentials (interpreted according to the SourceTracker results). Only the dispersal from outdoor air to indoor air (one direction) was considered. The bacterial results are based on our previous study [15]. Individual clip art images were downloaded from the open-source website Iconfont (http://www.iconfont.cn) and further customized

Based on our results, we present a summary of the dispersal potentials for bacteria and fungi for the different routes within households (Fig. 6). Microbes can be transferred between occupant skin and residential surfaces via human contact, with bacteria exhibiting a stronger dispersal potential compared to fungi. Although abundant cutaneous microbes can be aerosolized into indoor air, little evidence is found for the dispersal between indoor air and human skin or residential surfaces for both bacteria and fungi. Instead, the indoor airborne microbiome appears to be sourced from outdoor air regardless of ventilation strategy. Notably, indoor airborne bacterial communities are also significantly shaped by some household-specific bacteria from gut and oral cavity of human occupants [15]. In contrast, indoor airborne fungi are more susceptible to the geographic location and environmental factors, suggesting a stronger exchange between the indoor and outdoor fungal communities [72].

## Conclusions

In summary, our study provides evidence that occupants can transfer fungi to household surfaces via contact. Although almost half of the residential surfaces harbor occupants’ skin fungal fingerprint, fungi seem to be less readily transferred than their bacterial counterparts. Our study also shows that fungi appear to disperse farther in distance compared to bacteria during winter, and bacteria show dispersal limitation at a local scale. In addition, air currents, rather than building factors, appear to drive the spatial variation in the indoor airborne mycobiome in winter. This study provides important insights into the indoor transmission route of potential fungal allergens and pathogens, and the foundation to further study the complex relationships between indoor fungal exposure, occupant health, and the influence of outdoor environment.

## Additional files

Additional file 1: Text S1. Supplementary method file. (PDF 156 kb)

Additional file 2: Table S1. Categorization information of household occupancy and surface properties. (XLSX 28 kb)

Additional file 3: Table S2. Studies included in the source tracking analysis of indoor and outdoor air. (XLSX 29 kb)

Additional file 4: Figure S1. OTU rank-abundance curve. (PDF 431 kb)

Additional file 5: Figure S2. Venn-like representation of OTUs shared between the three sample types (air, skin, and surface) after rarefaction. Each circle represents one OTU and is colored by genus. The total number of OTUs is indicated in parentheses. (PDF 514 kb)

Additional file 6: Figure S3. The relative abundance of the skin-associated fungus *Malassezia* detected on frequently and less frequently touched surfaces. (PDF 1165 kb)

Additional file 7: Table S3. The indicator species detected in each sample type. (XLSX 41 kb)

Additional file 8: Table S4. Factors affecting the alpha-diversity of the indoor mycobiome. (XLSX 38 kb)

Additional file 9: Table S5. Binary Jaccard-based PERMANOVA test on factors affecting the indoor mycobiome. (XLSX 35 kb)

Additional file 10: Table S6. The cross-domain alpha-diversity comparison between bacteria and fungi in each type of samples. (XLSX 34 kb)

Additional file 11: Table S7. The cross-domain beta-diversity comparison between bacteria and fungi in each type of samples. (XLSX 34 kb)

Additional file 12: Figure S4. Distance-decay patterns of the airborne (A) bacterial and (B) fungal communities for households at the city-wide scale. (PDF 2418 kb)

Additional file 13: Figure S5. The contribution of the occupant skin and residential surface to the corresponding air community of each household. (A) The contribution of the skin community to the indoor air (which was sampled from four different rooms, *x*-axis) within each household (households ADMB and HHB without skin samples were excluded from the analysis). Correct prediction was made for households FT and QB. The percentage refers to the total contribution of each genus on the five skin sites to each air community. (B) The contribution of the surface community to the indoor air (*x*-axis) within each household. Correct prediction was only made for household MOS. The percentage refers to the total contribution of each genus on the eight different types of surfaces to each air community. (PDF 1006 kb)

Additional file 14: Figure S6. The contribution of the indoor air to the corresponding surface and skin community of each household. (A) The contribution of the air community to the residential surface (*x*-axis) within each household. The percentage refers to the total contribution of each genus in the air of the four rooms to each surface community. Correct prediction was only made for household TY. (B) The contribution of the air community to each skin site (right *y*-axis) of the occupants (*x*-axis) within each household. The percentage refers to the total contribution of each genus in the air of the four rooms to each skin community. No correct prediction was made in the analysis. (PDF 1317 kb)

Additional file 15: Table S8. The contributor OTUs detected exclusively on skin or surfaces. (XLSX 35 kb)

Additional file 16: Figure S7. SourceTracker prediction of the contribution of outdoor air (Beijing, China and Berkeley, USA) to HK indoor airborne mycobiome. (A) Closed-reference OTU picking strategy and (B) Open-reference OTU picking strategy are plotted (PDF 1149 kb)
