# Supplementary material for: City-scale distribution and dispersal routes of mycobiome in residences
Source: Microbiome. 2017 Oct 4;5:131. doi: 10.1186/s40168-017-0346-7 (PMC5628474; doi:10.1186/s40168-017-0346-7)
Supplement: Supplementary file 1 — Supplementary method file. (PDF 156 kb) [file 40168_2017_346_MOESM1_ESM.pdf]

Supplementary methods

Study locations and sample collection

The 19 households in this study are distributed spatially throughout urban and rural areas of HK. None of the households are located within the same building (Fig. 1). Briefly, the households were selected to cover a range of building designs and architecture layouts, and no mold or water flooding issues had been reported in the months leading up to sampling. A total of 428 household and occupant skin samples were collected during the month of January in 2014, including 76 air samples from four room types (living room, bedroom, kitchen, and toilet), 200 skin samples from five different body sites (forehead, left and right forearm, and left and right palm) of 40 healthy Chinese individuals (skin samples from households ADMB and HHB were not available), and 152 surface samples from eight different residential surfaces (bed headboard, blanket, shower curtain, toilet flush button, remote control, fridge door seal, kitchen ventilator, and TV screen).

Air samples were collected as described previously [1]. Briefly, airborne particles were impacted onto an autoclaved 25 mm cellulose nitrate filter (pore size 0.2 μm; GE HealthCare, UK) with a Sioutas Cascade Impactor (SKC Inc., Eighty Four, PA, USA) and a D-plate accelerator (collects particles > 0.25 μm in diameter) at a flow rate of 9 L/min for 12 hours per sampling day (~6.5 m^3 total volume). Skin and residential surfaces were swabbed for 15 seconds with the autoclaved cotton tips which were pre-moistened in a sterile swab solution (0.15 M NaCl, 0.1% Tween 20) [2]. All samples were stored in –80 °C within one hour of sampling until genomic DNA (gDNA) extraction. Sterile filters and swabs that had not been used in sampling were included as negative blank controls to facilitate contaminant detection and removal (see below).

DNA extraction, library preparation and sequencing

gDNA was extracted using the PowerSoil DNA Isolation Kit (MO BIO Laboratories, Inc., Carlsbad, CA, USA) and performed as described previously [3]. Purified gDNA was sent to SeqMatic (Fremont, CA, USA) for PCR amplification, sequence library preparation and sequencing as described previously [4] with minor modifications. Amplification was performed to target the first fungal internal transcribed spacer (ITS1) region with the forward (ITS1-18S*fw*: 5’-GTAAAAGTCGTAACAAGGTTT C-3’) and reverse primers (ITS1-5.8S*rv*: 5-'GTTCAAAGAYTCGATGATTCAC-3’) [5], with an expected amplicon size of 350 to 450 bp.

The ITS1 amplicons for Illumina MiSeq sequencing were generated using a two-step amplification procedure. The first round of amplification was carried out in triplicate. Each 10 μL reaction mix contained 7 μL of TailorMix 2× SYBR Green qPCR Master Mix (SeqMatic, Fremont, CA, USA), 1 μM of each primer and 1 μL of template DNA. Thermal and cycling conditions were as follows: an initial 10-min denaturation at 95°C before undergoing 35 cycles of 95°C for 30 s, 50°C for 1 min and 60°C for 1 min, and a final 10-min extension at 60°C. Amplicons in triplicate were pooled, purified with the DNA/RNA Purification Beads (SeqMatic, Fremont, CA, USA) and re-suspended in 12 μL of 10 mM Tris (pH 8.5). The second round of indexing-PCR was performed in a 20-μL reaction volume containing 7.5 μL of purified amplicon, 10.5 μL of TailorMix 2× SYBR Green qPCR Master Mix (SeqMatic, Fremont, CA, USA), 10 μM of each forward (Index Primer 1) and reverse (Index Primer 2) indexing primer. Following an initial 10-min denaturation at 95°C, each sample was amplified by 8 cycles of 95°C for 15 s, 55°C for 1 min and 72°C for 1.5 min and a final 10-min extension at 72°C. Both rounds of PCR amplification were conducted on the 7500 Fast Real-Time PCR System (Applied Biosystems, Foster City, CA, USA).

Equal volume of each indexed amplicon was pooled and purified by agarose gel electrophoresis. Libraries were prepared using the Illumina MiSeq Reagent Kit v2, and the

final library was quantified with the 2200 TapeStation (Agilent, Santa Clara, CA, USA) and sequenced on the Illumina MiSeq platform to generate 250 bp pair-end reads. Multiplexing/barcoding was performed through the Indexing PCR where each sample was assigned a unique combination of i5 + i7 nextera index. Samples and their sequencing data were computationally demultiplexed after the sequencing run.

OTU formation and indicator taxa

Forward and reverse reads were merged using the "-fastq_mergepairs" command of USEARCH (version 9.0.2132) [6]. Quality control was performed using the "fastq_filter" command of USEARCH with a maximum expected error of 0.5 error per read. Reads were trimmed to a uniform length of 300 bp and shorter reads were discarded. Dereplicated reads were clustered into operational taxonomic units (OTUs) based on 97% similarity threshold following the UPARSE pipeline [7], with the "cluster_otu" command in USEARCH. In addition to the initial detection of chimeric OTUs by the built-in *de novo* filters of USEARCH, an additional round of chimera filtering were carried out using the "uchime_ref" command in USEARCH [8], based on the UCHIME ITS1 reference database (11 March 2015 version) [9]. Taxonomic information was assigned to OTUs with UCLUST using the QIIME (version 1.9.1) [10] script "assign_taxonomy.py" with two reference databases (see section "*Taxonomic classification with fungal reference databases*" for details). For contaminant removal, OTUs belonging to taxonomic lineages (i.e. the top-down taxonomic hierarchical classification from the rank of kingdom to the rank of species) present at an average of greater than 3% relative abundance in the negative blank controls were removed from subsequent samples.

For indicator species analysis [11], only OTUs with a relative abundance greater than 1% in at least one sample were included. Indicator taxa were identified using the "indval" function in the R package "labdsv" [12]. An indicator value threshold of greater than 0.5 was

applied and significance was determined based on a False Discovery Rate (FDR) [13] corrected $p < 0.05$ after 1,000 permutations.

Taxonomic classification with fungal reference databases

Given that the UNITE reference database [14] is the gold standard widely used in mycobiome analyses across different ecosystems [15, 16], taxonomic classification based on the UNITE ITS1 dataset was first performed using the UCLUST algorithm with a minimum similarity threshold of 0.90. Although three phyla, 17 classes, 49 orders, 100 families, and 180 genera were identified, an 82.0% of reads remained unclassified at the genus level. Moreover, while about half of the samples originated from skin, *Malassezia*, which has been reported as the most common skin fungus [4, 17], was less abundant than other genera in our occupant skin samples. In order to obtain taxonomic information for the reads unclassified by UNITE at the genus level, we further classified those reads against a curated ITS database which was optimized for analyzing the skin mycobiome [5] using the same algorithm as above. Through this, the proportion of unclassified reads at the genus level was reduced from 82.0% to 17.8%, with a corresponding increase from 180 to 276 in the total number of genera. Reads that could not be assigned at the genus level with the curated database would adopt the taxonomic information (from phylum to family) of the curated database.

The nonparametric Mann-Whitney (MW, two groups) [18] and Kruskal-Wallis (KW, at least three groups) tests [19] were used to determine the significance of the relative abundance of a given genus between groups. KW post-hoc test was performed for pairwise comparisons using the "kruskalmc" function in the R package "pgirmess" [20] when an overall statistically significant difference was observed (KW test, $p < 0.05$).

Influences of building and location factors on community richness and structure

Samples were rarefied to a depth of 1,058 reads per sample before community analysis and those with fewer than 1,058 reads were discarded, reducing the number of samples to 383.

The average Good's coverage across the remaining samples was 99.1 ± 0.5% (one standard deviation), indicating that the rarefaction depth adopted was sufficient to capture the majority of OTU richness. The building and location factors considered include sample type (air/skin/surface), individual (for skin samples), household, room (for air and surface samples), area (residential surface for surface samples, body site for skin samples), human touch frequency (high/low, for surface samples), surface deposition potential for aerosol and dust (high/low, for surface samples), household occupancy level (high/low, for air and surface samples), and temperature, relative humidity as well as building ventilation type (natural/mechanical) during most of the sampling time (for air samples). The categorization information of household occupancy and surface properties as well as the sample size for each category are listed in Table S1.

Alpha-diversity metrics of community richness (observed number of OTUs and Chao1 [21]) and diversity (Shannon [22] and Simpson [23]), and beta-diversity distances between samples (abundance-based Bray-Curtis dissimilarity [24] and incidence-based Binary Jaccard distance [25]) were calculated using the scripts "alpha_diversity.py" and "beta_diversity.py" in QIIME [10] with default settings. The significant effects of building and location factors on community richness and diversity were determined using the analysis of variance (ANOVA) by comparing the linear mixed-effects models with and without the factor of interest in the R package "lme4" [26]. Permutational multivariate analysis of variance (PERMANOVA) was carried out using the "adonis" function in the R package "vegan" [27] to examine the influence of different factors on community structure and composition, with significance determined through 999 random permutations.

Cross-domain bacterial and fungal analysis

To determine the correlation between bacterial and fungal community richness and composition, alpha- (observed number of OTUs and Chao1) and beta- (Bray-Curtis

dissimilarity and Binary Jaccard distance) diversity were calculated for our previously reported bacterial dataset [1]. Cross-domain alpha- and beta-diversity correlations, Spearman's correlation, and linear regression were computed in R. Effect size was further calculated to assess the extent of correlation for the significant groups as described previously [4].

Dispersal prediction model

SourceTracker [28] was used to test the dispersal potentials between occupants' skin, residential surfaces and indoor air. Only OTUs present in at least 10% of the samples were included for analysis. For dispersal routes between air and surface samples, SourceTracker predictions were generated for all 19 households. For dispersal routes including skin either as a source or sink, SourceTracker predictions were only performed for 17 households (two households without skin samples were excluded). Furthermore, separate predictions were made for routes involving air samples based on high/low occupancy and surface samples based on high/low touch, high/low deposition or high/low occupancy. For the sink community in one specific household, the contributions of the source communities from all households were tested. The prediction was considered successful if the source community with the greatest contribution to the sink community was from the same household. However, a relatively weaker contribution compared to other households did not necessarily mean that no dispersal occurred. The results were presented as an accuracy rate, the ratio between the number of successful predictions and total predictions [1].

In order to further study which OTUs could be transferred between air, skin and surfaces, and the extent to which the OTUs contributed to shaping the sink community, the relative contribution rates of the source OTUs to the correct sink communities were assessed for all households. Here, source OTUs are referred to as contributor OTUs if they contribute to the corresponding sink communities within the same household. The proportions of contributor OTUs were aggregated at the genus level.

Comparison with outdoor air samples

To test the hypothesis that outdoor air was the dominant source for the indoor mycobiome, fungal community data from outdoor air samples from Beijing, China [29] and Berkeley, USA [30] were retrieved as surrogate sources as there is no similar mycobiome dataset of outdoor air in HK. As with this study, these two studies targeted the ITS1 region and used an Illumina MiSeq platform for sequencing (Additional file 2: Table S2). To allow comparison with this study, the raw sequences from the indoor and outdoor air samples were processed using the UPARSE pipeline described above. Briefly, the forward raw reads were filtered with a maximum error rate of 0.5 error per read and trimmed to a uniform length of 240 bp. After quality filtering, a total of 3,232,189 reads were retained for OTU clustering. Closed-reference OTU picking was executed with the QIIME script "pick_closed_reference_otus.py" with default settings to pick OTUs against a full "UNITE+INSD" database (31 Jan 2016 version) using UCLUST at 97% similarity, with any reads that failed to hit the reference discarded from OTU clustering. Through this, 4,110 non-singleton OTUs comprising 1,866,048 reads were retained. Since the number of OTUs recovered from the above three studies were constrained by the coverage of the reference database, an additional round of analysis was performed with the script "pick_open_reference_otus.py" in QIIME with default parameters, where reads that did not hit the reference sequence collection in the closed-reference workflow were subsequently clustered *de novo*. Following the open-reference OTU picking strategy, a total of 12,748 non-singleton OTUs comprising 3,224,519 reads were retained.

SourceTracker analysis was performed to assess the contribution of outdoor air samples from Beijing and Berkeley as the potential sources, with HK indoor air samples as sinks. OTU tables generated after the above two OTU picking strategies were filtered respectively with

"pick_otus_from_otu_table.py" and OTUs presented in less than 10% of all samples were removed from the SourceTracker analysis.

Distance-decay biogeographic pattern

To test the hypothesis that a distance-decay relationship exists in the indoor airborne mycobiome, correlations between the indoor bacterial or fungal community dissimilarity and geographic distance were studied using the Binary Jaccard distance metric. The geographic distance between any two households was measured based on the point-to-point straight-line distance using the "Measure Distance" function in Google Map. Mantel test [31] was performed to test the significance of distance-decay pattern using the R package "ade4" [32], with significance determined through 9,999 random permutations.

References

1. Wilkins D, Leung MHY, Lee PKH. Indoor air bacterial communities in Hong Kong households assemble independently of occupant skin microbiomes. Environ Microbiol. 2016;18(6):1754-63.

2. Fierer N, Hamady M, Lauber CL, Knight R. The influence of sex, handedness, and washing on the diversity of hand surface bacteria. Proc Natl Acad Sci U S A. 2008;105(46):17994-9.

3. Leung MHY, Wilkins D, Li EKT, Kong FKT, Lee PKH. Indoor-air microbiome in an urban subway network: diversity and dynamics. Appl Environ Microbiol. 2014;80(21):6760-70.

4. Leung MHY, Chan KCK, Lee PKH. Skin fungal community and its correlation with bacterial community of urban Chinese individuals. Microbiome. 2016;4(1):46.

5. Findley K, Oh J, Yang J, Conlan S, Deming C, Meyer JA et al. Topographic diversity of fungal and bacterial communities in human skin. Nature. 2013;498(7454):367-70.

6. Edgar RC. Search and clustering orders of magnitude faster than BLAST. Bioinformatics. 2010;26(19):2460-1.

7. Edgar RC. UPARSE: highly accurate OTU sequences from microbial amplicon reads. Nat Methods. 2013;10(10):996-8.

8. Edgar RC, Haas BJ, Clemente JC, Quince C, Knight R. UCHIME improves sensitivity and speed of chimera detection. Bioinformatics. 2011;27(16):2194-200.

9. Nilsson RH, Tedersoo L, Ryberg M, Kristiansson E, Hartmann M, Unterseher M et al. A comprehensive, automatically updated fungal ITS sequence dataset for reference-based chimera control in environmental sequencing efforts. Microbes Environ. 2015;30(2):145.

10. Caporaso JG, Kuczynski J, Stombaugh J, Bittinger K, Bushman FD, Costello EK et al. QIIME allows analysis of high-throughput community sequencing data. Nat Methods. 2010;7(5):335-6.

11. Dufrene M, Legendre P. Species assemblages and indicator species: the need for a flexible asymmetrical approach. Ecol Monogr. 1997;67(3):345-66.

12. Roberts D. Labdsv: ordination and multivariate analysis for ecology. R package version 1.7–0. (2015).

13. Benjamini Y, Hochberg Y. Controlling the false discovery rate: a practical and powerful approach to multiple testing. J R Stat Soc Series B (Methodological). 1995;57(1):289-300.

14. Kõljalg U, Larsson KH, Abarenkov K, Nilsson RH, Alexander IJ, Eberhardt U et al. UNITE: a database providing web-based methods for the molecular identification of ectomycorrhizal fungi. New Phytol. 2005;166(3):1063-8.

15. Li W, Wang MM, Wang XG, Cheng XL, Guo JJ, Bian XM et al. Fungal communities in sediments of subtropical Chinese seas as estimated by DNA metabarcoding. Sci Rep. 2016;6:26528.

16. Amend AS, Seifert KA, Samson R, Bruns TD. Indoor fungal composition is geographically patterned and more diverse in temperate zones than in the tropics. Proc Natl Acad Sci U S A. 2010;107(31):13748-53.

17. Findley K, Oh J, Yang J, Conlan S, Deming C, Meyer JA et al. Human skin fungal diversity. Nature. 2013;498(7454):367.

18. Gehan EA. A generalized Wilcoxon test for comparing arbitrarily singly-censored samples. Biometrika. 1965;52(1-2):203-24.

19. Breslow N. A generalized Kruskal-Wallis test for comparing K samples subject to unequal patterns of censorship. Biometrika. 1970;57(3):579-94.

20. Giraudoux P, Giraudoux MP. Package 'pgirmess'. R package version 1.6.7. (2017).

21. Chao A. Estimating the population size for capture-recapture data with unequal catchability. Biometrics. 1987;43:783-91.

22. Shannon CE, Weaver W. The mathematical theory of communication. University of Illinois press. Urbana. 1998.

23. Simpson EH. Measurement of diversity. Nature. 1949;163:688.

24. Bray JR, Curtis JT. An ordination of the upland forest communities of southern Wisconsin. Ecol Monogr. 1957;27(4):325-49.

25. Jaccard P. Contribution au problème de l; immigration post-glacière de la flore alpine: etude comparative de la flore alpine du massif du Wildhorn, du haut bassin du Trient et de la haute vallée de Bagnes. 1900.

26. Bates D, Mächler M, Bolker B, Walker S. Fitting linear mixed-effects models using lme4. J Stat Softw. 2014;67:1-48.

27. Oksanen J, Blanchet F, Friendly M, Kindt R, Legendre P, Mcglinn D et al. vegan: community ecology package. R package version 2.4-2. (2017).

28. Knights D, Kuczynski J, Charlson ES, Zaneveld J, Mozer MC, Collman RG et al. Bayesian community-wide culture-independent microbial source tracking. Nat Methods. 2011;8(9):761-3.

29. Yan D, Zhang T, Su J, Zhao L-L, Wang H, Fang X-M et al. Diversity and composition of airborne fungal community associated with particulate matters in Beijing during haze and non-haze days. Front Microbiol. 2016;7:487.

30. Adams RI, Bhangar S, Pasut W, Arens EA, Taylor JW, Lindow SE et al. Chamber bioaerosol study: outdoor air and human occupants as sources of indoor airborne microbes. PLoS ONE. 2015;10(5):e0128022.

31. Mantel N. The detection of disease clustering and a generalized regression approach. Cancer Res. 1967;27(2 Part 1):209-20.

32. Chessel D, Dufour A-B, Dray S. Analysis of ecological data: exploratory and euclidean methods in environmental sciences. R package version 1.4-17. (2011).
