# Supplementary figures and images for: City-scale distribution and dispersal routes of mycobiome in residences
Source: Microbiome. 2017 Oct 4;5:131. doi: 10.1186/s40168-017-0346-7 (PMC5628474; doi:10.1186/s40168-017-0346-7)

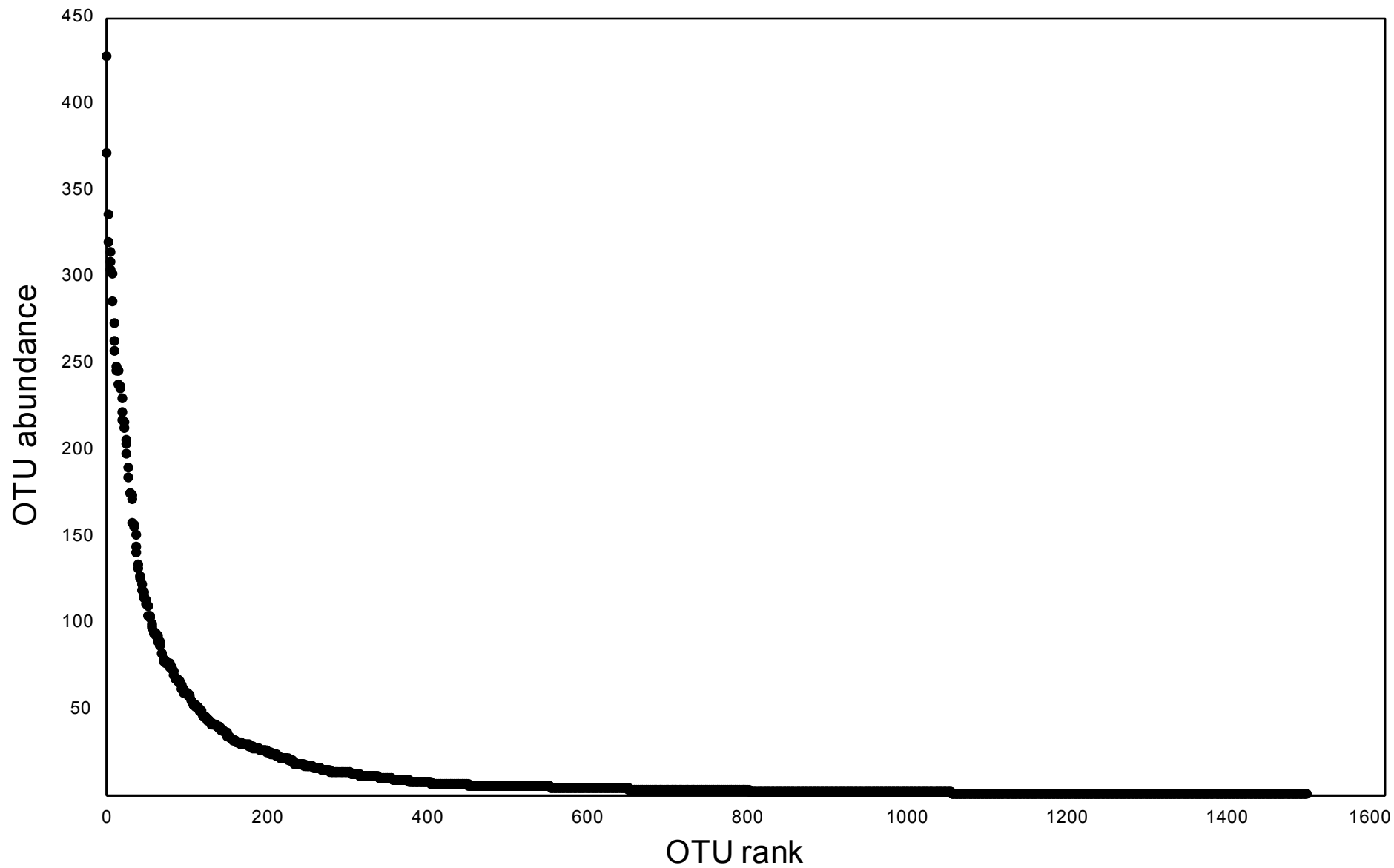

Supplement: Supplementary file 4 — OTU rank-abundance curve. (PDF 431 kb) [file 40168_2017_346_MOESM4_ESM.pdf]

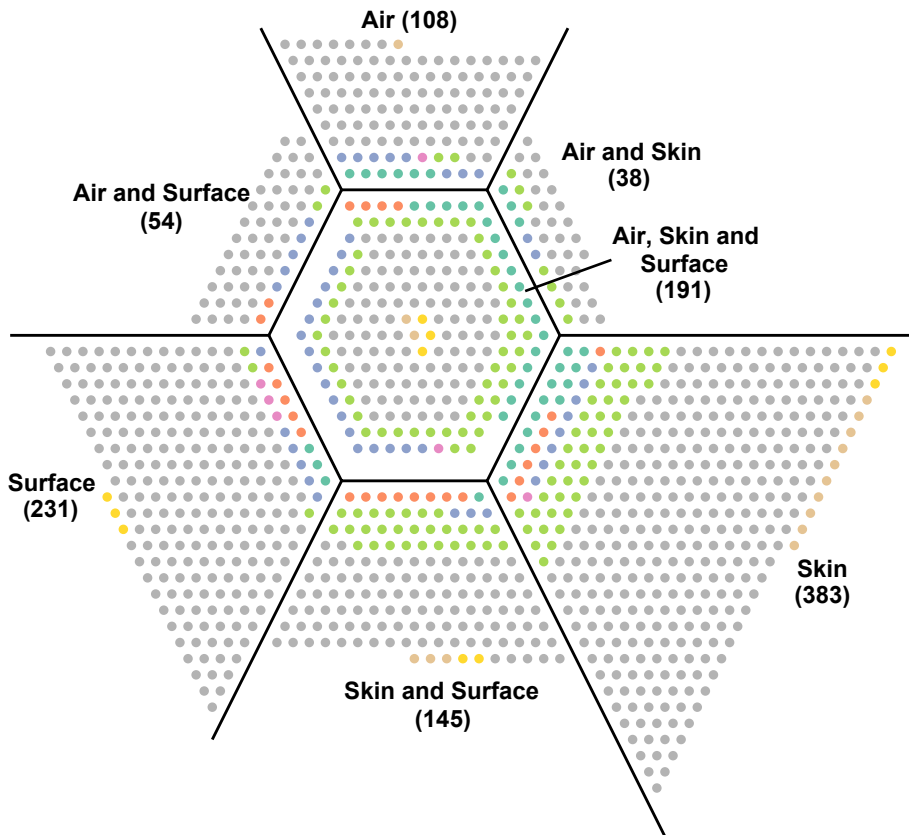

Supplement: Supplementary file 5 — Venn-like representation of OTUs shared between the three sample types (air, skin, and surface) after rarefaction. Each circle represents one OTU and is colored by genus. The total number of OTUs is indicated in parentheses. (PDF 514 kb) [file 40168_2017_346_MOESM5_ESM.pdf]

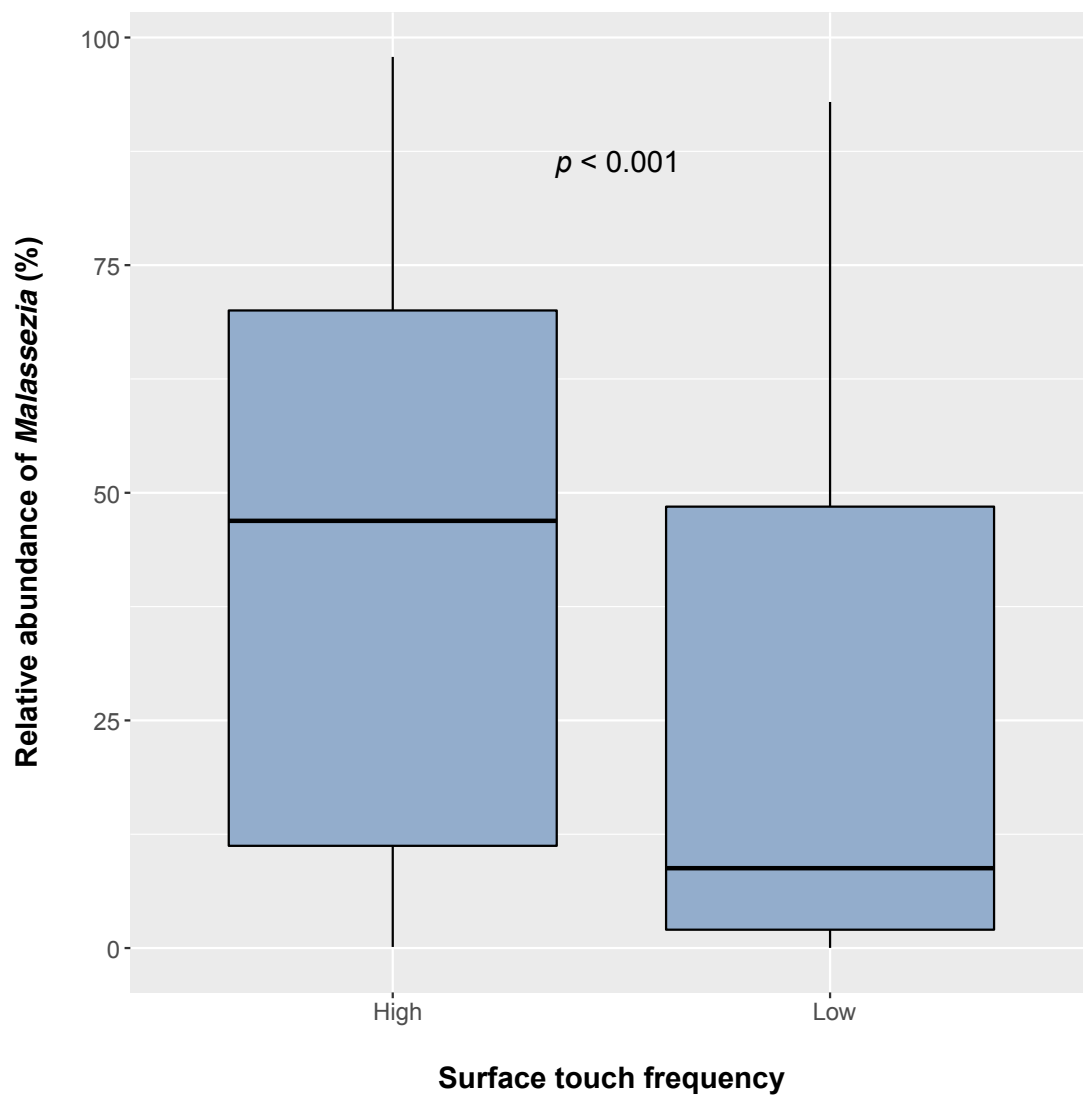

Supplement: Supplementary file 6 — The relative abundance of the skin-associated fungus Malassezia detected on frequently and less frequently touched surfaces. (PDF 1165 kb) [file 40168_2017_346_MOESM6_ESM.pdf]

**A**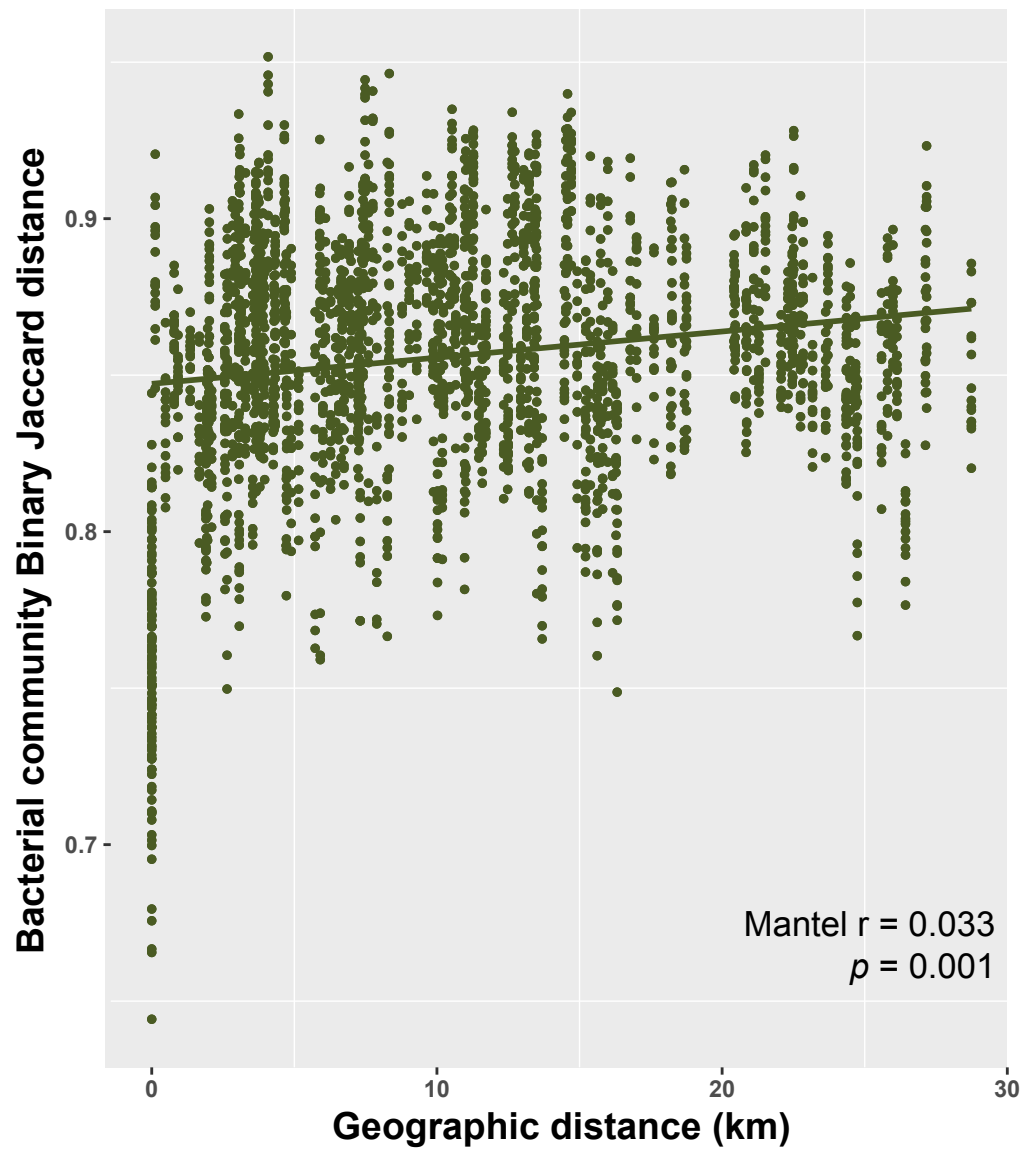**B**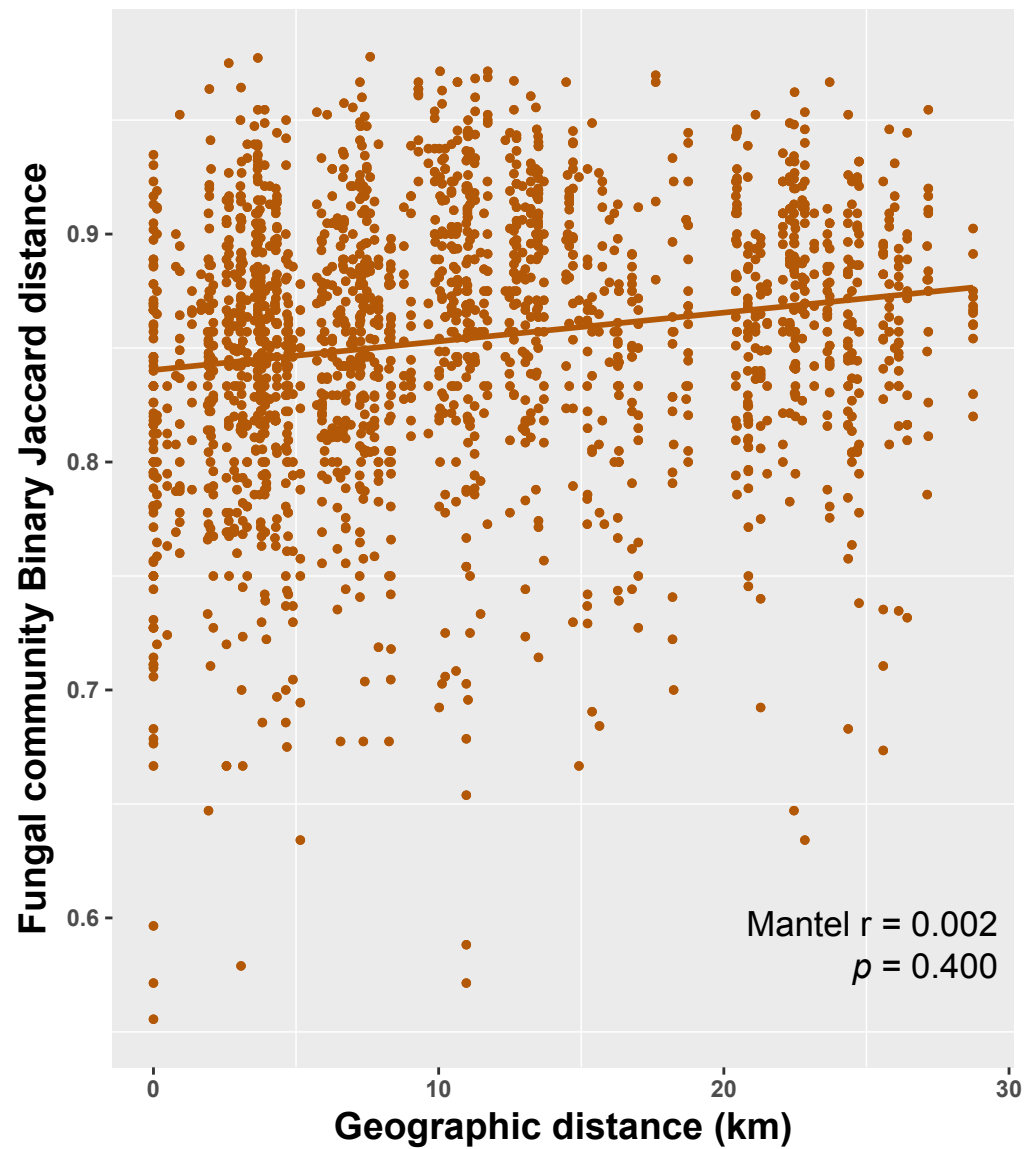

Supplement: Supplementary file 12 — Distance-decay patterns of the airborne (A) bacterial and (B) fungal communities for households at the city-wide scale. (PDF 2418 kb) [file 40168_2017_346_MOESM12_ESM.pdf]

**A**

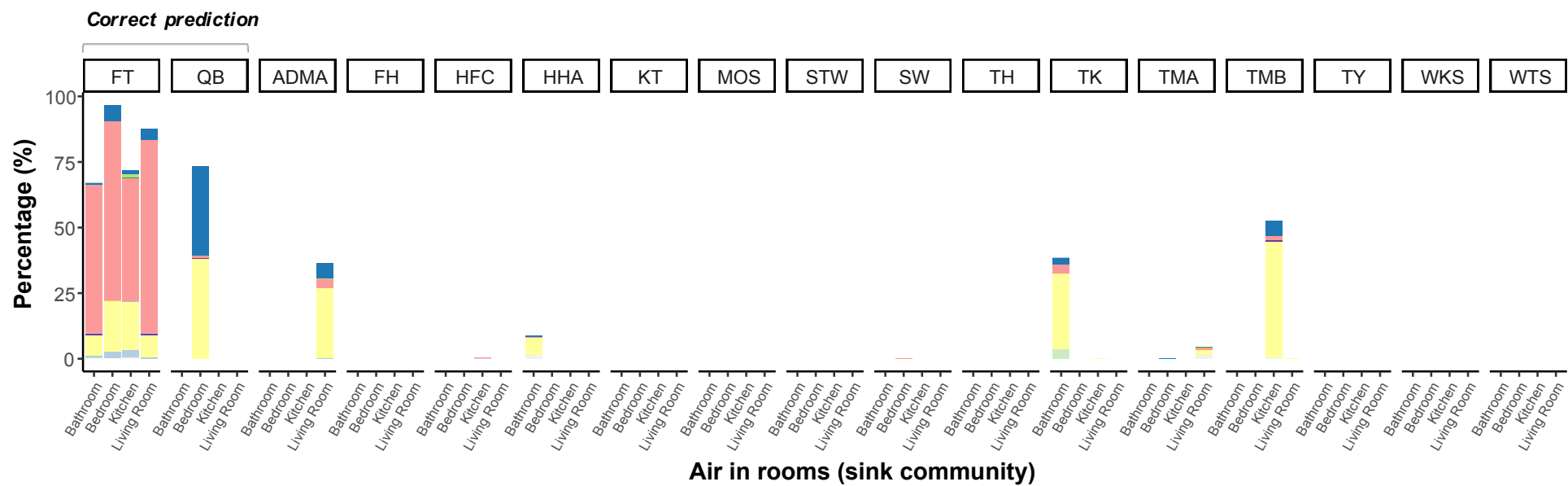

B

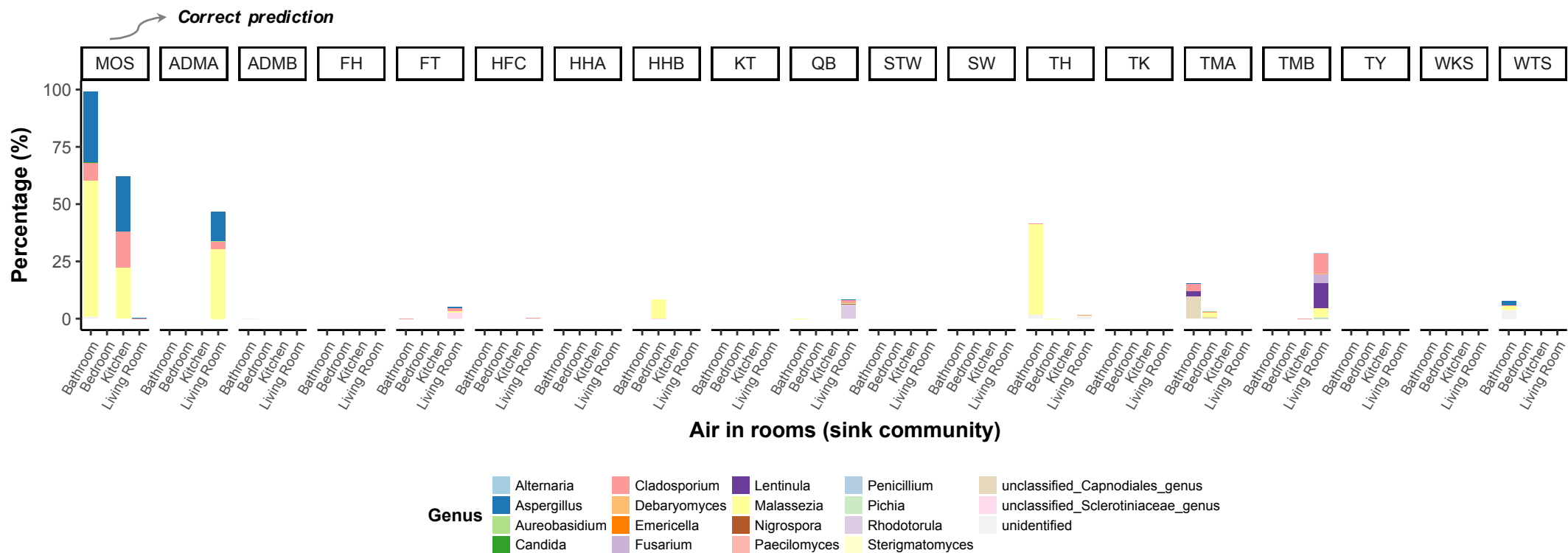

Supplement: Supplementary file 13 — The contribution of the occupant skin and residential surface to the corresponding air community of each household. (A) The contribution of the skin community to the indoor air (which was sampled from four different rooms, x-axis) within each household (households ADMB and HHB without skin samples were excluded from the analysis). Correct prediction was made for households FT and QB. The percentage refers to the total contribution of each genus on the five skin sites to each air community. (B) The contribution of the surface community to the indoor air (x-axis) within each household. Correct prediction was only made for household MOS. The percentage refers to the total contribution of each genus on the eight different types of surfaces to each air community. (PDF 1006 kb) [file 40168_2017_346_MOESM13_ESM.pdf]

A

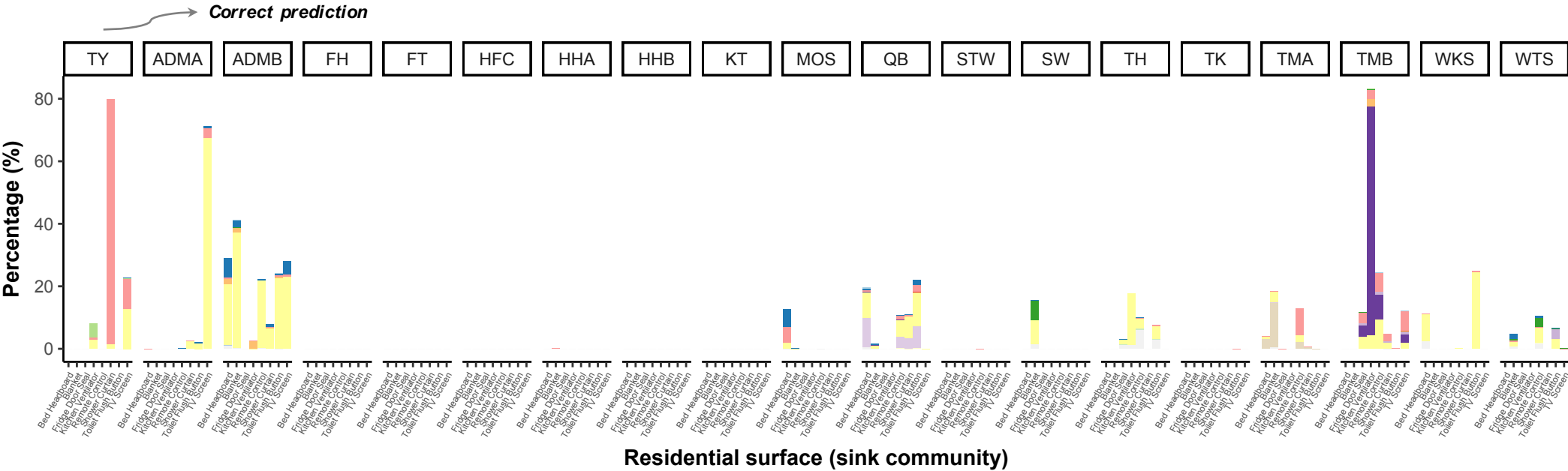

B

False prediction

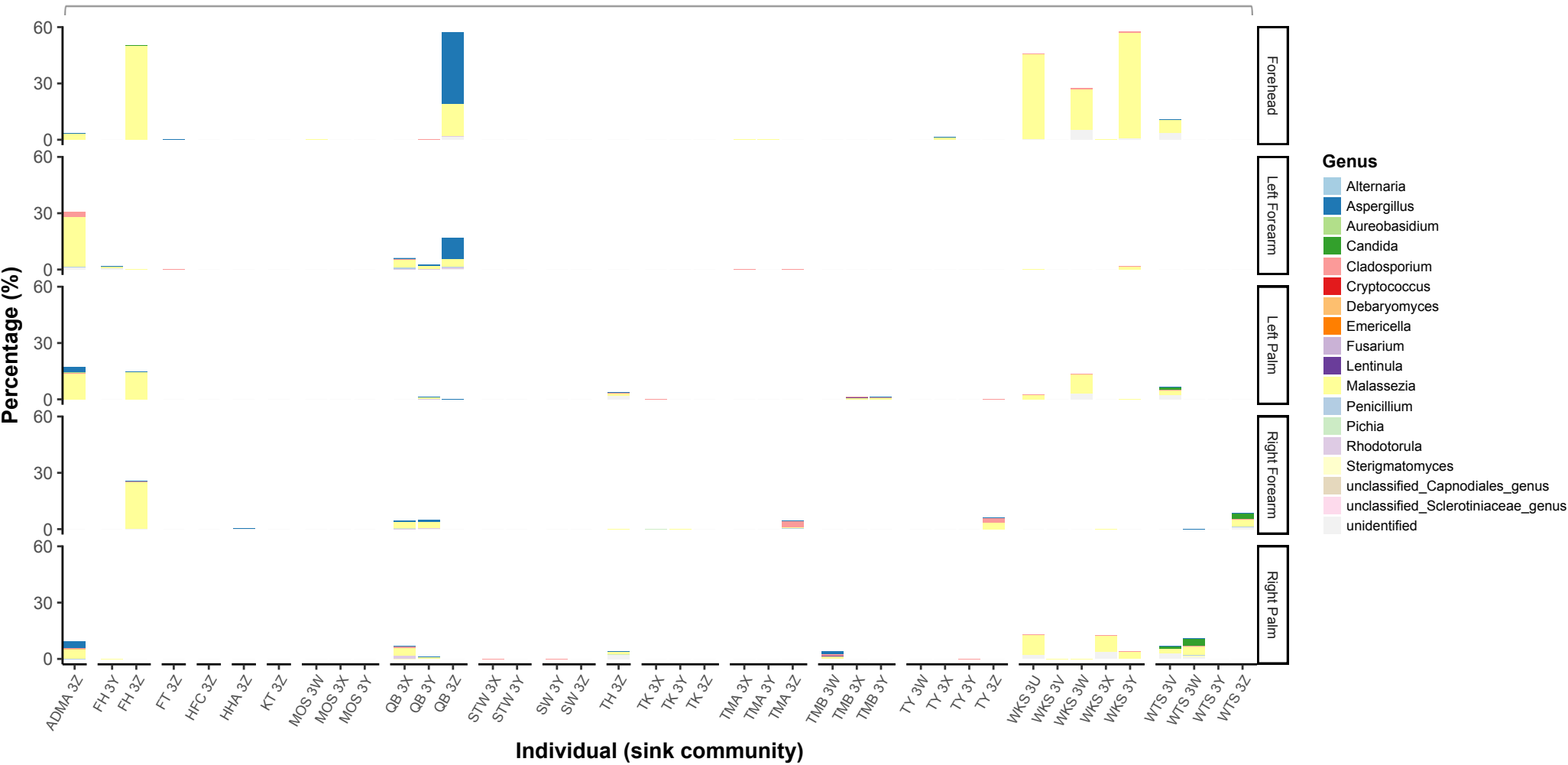

Supplement: Supplementary file 14 — The contribution of the indoor air to the corresponding surface and skin community of each household. (A) The contribution of the air community to the residential surface (x-axis) within each household. The percentage refers to the total contribution of each genus in the air of the four rooms to each surface community. Correct prediction was only made for household TY. (B) The contribution of the air community to each skin site (right y-axis) of the occupants (x-axis) within each household. The percentage refers to the total contribution of each genus in the air of the four rooms to each skin community. No correct prediction was made in the analysis. (PDF 1317 kb) [file 40168_2017_346_MOESM14_ESM.pdf]

A

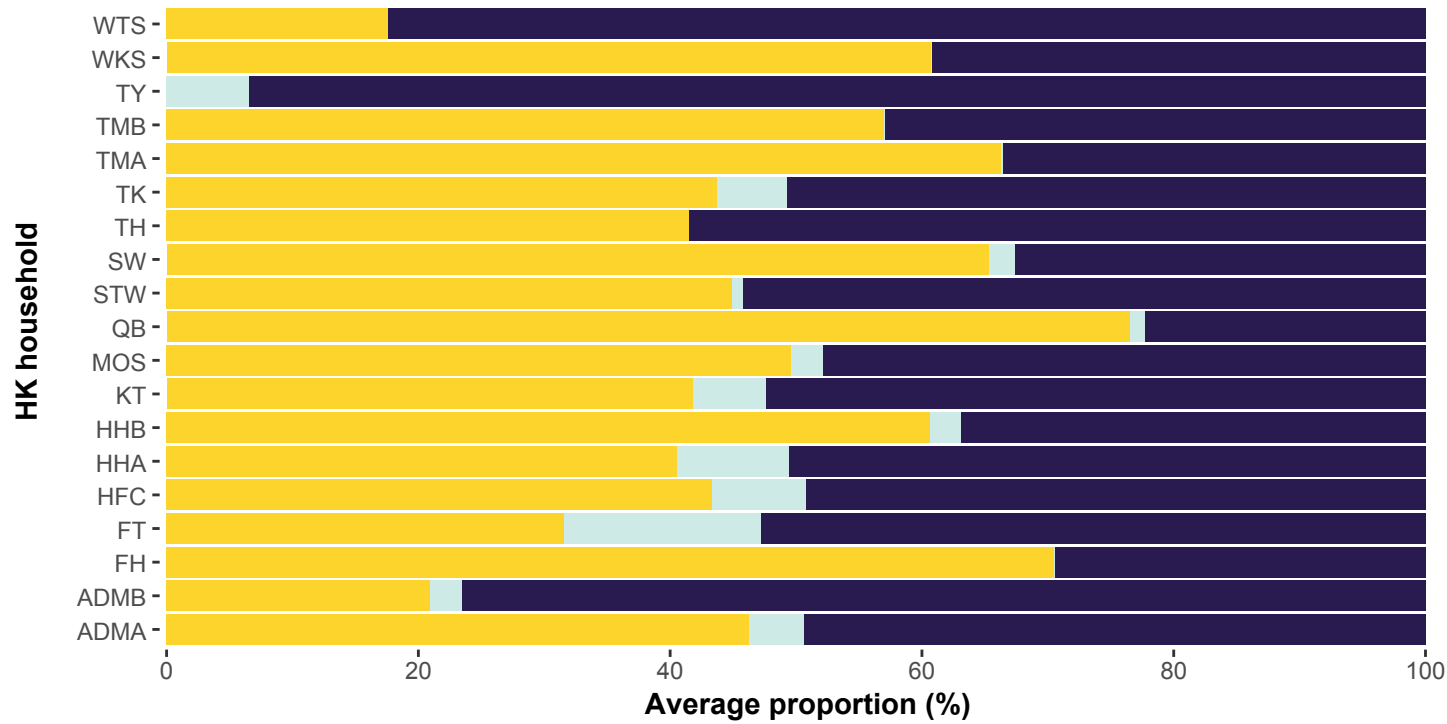

B

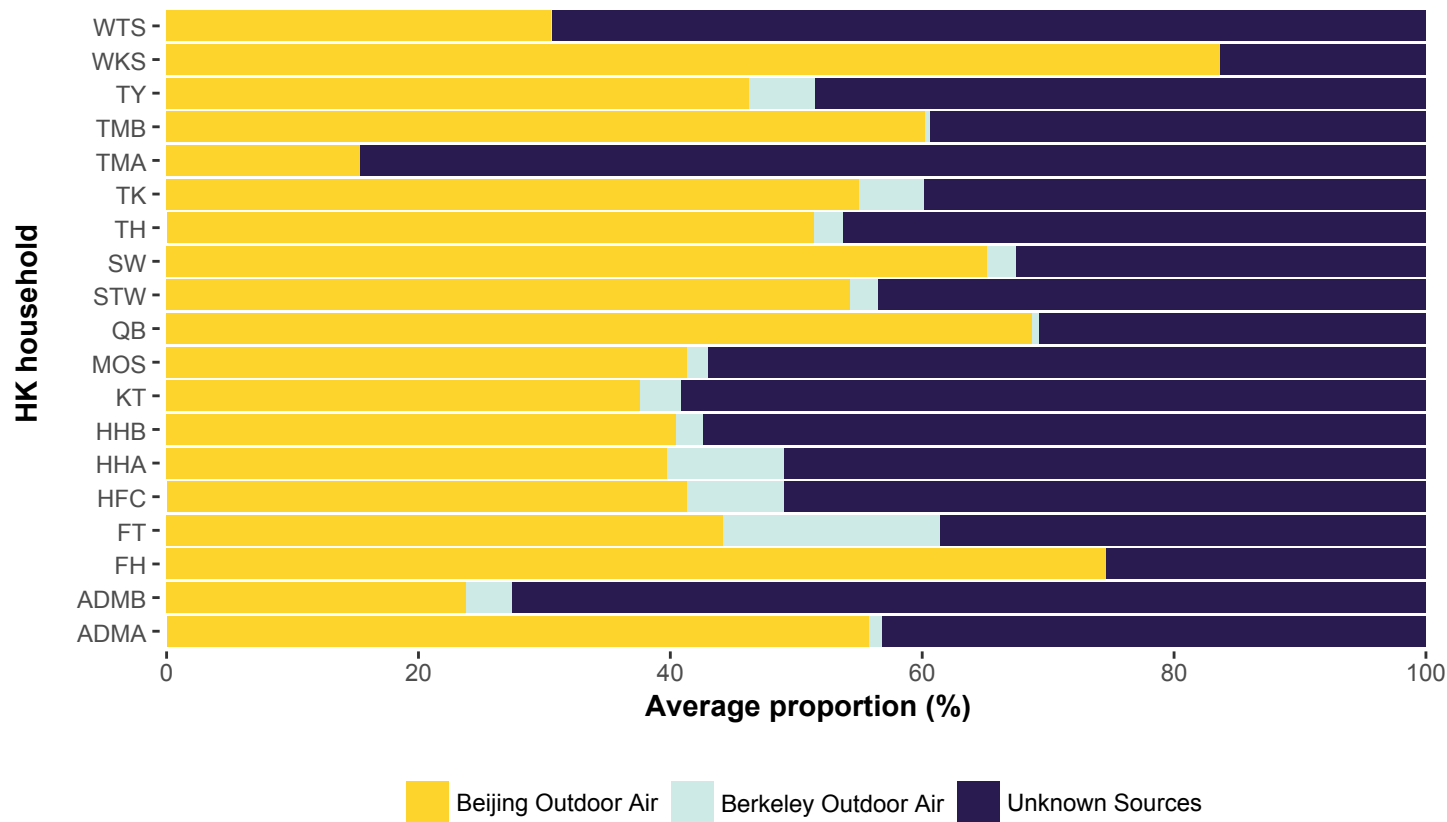

Supplement: Supplementary file 16 — SourceTracker prediction of the contribution of outdoor air (Beijing, China and Berkeley, USA) to HK indoor airborne mycobiome. (A) Closed-reference OTU picking strategy and (B) Open-reference OTU picking strategy are plotted (PDF 1149 kb) [file 40168_2017_346_MOESM16_ESM.pdf]
